# Paediatric tuberculosis during universal and selective *Bacillus* Calmette–Guérin vaccination policy: a nationwide population-based retrospective study, Finland, 1995–2015

**DOI:** 10.2807/1560-7917.ES.2021.26.11.1900711

**Published:** 2021-03-18

**Authors:** Antti Kontturi, Satu Kekomäki, Hanna Soini, Jukka Ollgren, Eeva Salo

**Affiliations:** 1 Doctoral Programme in Population Health, University of Helsinki, Helsinki, Finland; 2 Department of Pediatrics, Children’s Hospital, University of Helsinki and Helsinki University Hospital, Helsinki, Finland; 3 Department of Health Security, National Institute for Health and Welfare, Helsinki, Finland

**Keywords:** tuberculosis, paediatric tuberculosis, BCG, public health, vaccine, vaccination policy

## Abstract

**Introduction:**

In 2006, the *Bacillus* Calmette–Guérin (BCG) vaccination policy in Finland changed from universal to selective.

**Aim:**

We assessed the impact of the policy change on tuberculosis (TB) morbidity in children under 5 years and epidemiological trends of paediatric TB in Finland.

**Methods:**

We conducted a nationwide, population-based, retrospective registry study of all newly diagnosed active TB cases younger than 15 years in Finland from 1995 to 2015 by linking data from the National Infectious Diseases Register, Finnish Care Register for Health Care, medical patient records and Finnish Population Information System. We compared the TB incidence rate ratio of under 5 year-olds with universal and selective BCG vaccinations with a Poisson log-linear model and analysed incidence trends among those younger than 15 years with a negative binomial model.

**Results:**

We identified 139 paediatric TB cases: 50 native (including 24 second-generation migrants) and 89 foreign-born children. The TB rate of under 5 year-olds remained stable after changing to selective BCG vaccination (incidence rate ratio (IRR): 1.3; 95% confidence interval (CI): 0.7–2.3). TB rate in the native population under 15 years increased slightly (IRR = 1.06; 95% CI: 1.01–1.11).

**Discussion:**

Paediatric TB cases in Finland were concentrated in families with migrant background from high-TB incidence countries. The native TB morbidity in under 5-year-olds did not increase after the BCG policy revision, suggesting that selective vaccinations can prevent TB in the most vulnerable age group in low-incidence settings. Second-generation migrants under 15 years in Finland with high TB risk are probably increasing.

## Introduction

The World Health Organization (WHO) declared tuberculosis (TB) a global emergency more than 25 years ago. However, TB remains a major public health concern and a leading cause of death from infection [1]. Young children are especially vulnerable to TB and severe disease [2]. In children, the incubation period from primary infection to disease is usually less than a year; childhood TB signals recent transmission and is a good indicator of the effectiveness of national TB control [3]. Nevertheless, epidemiological surveillance is generally focused on the adult population and the paediatric TB burden is often neglected.

Infant *Bacillus* Calmette–Guérin (BCG) vaccinations prevent serious TB in children effectively [2,4]. Universal BCG vaccinations are still common in Europe [5]. The International Union Against Tuberculosis and Lung Disease and the WHO are, however, encouraging countries with declining TB incidence to consider discontinuation of universal BCG vaccinations [6,7]. The BCG policy revision is a major effort for national TB control programmes and understanding the implications is imperative [8]. The evidence of a threshold incidence for safe discontinuation of universal BCG is limited [8]. The initial studies evaluated BCG policy revisions that took place over 30 years ago and the landscape of TB in Europe has since changed drastically [9,10]. Recent nationwide population-based evaluations of universal BCG discontinuation are scarce, especially regarding BCG vaccination at birth, which is the WHO recommendation and the most common universal policy in Europe [5,7].

Since 2001, the overall TB incidence in Finland has been lower than 10 per 100,000 population [11]. In 2006, the BCG policy changed from universal at birth to selective vaccinations of children at high TB risk only [12]. Consequently, the BCG coverage of infants dropped from more than 98% to an estimated 6–10% and the ensuing birth cohorts have grown up predominantly without BCG protection [13,14]. Simultaneously, immigration from countries with high TB incidence has caused a major transition of TB morbidity from the old indigenous to the young foreign-born population [11]. While TB cases among the native adult population have decreased, the migrant population and proportion of non-native TB cases among all cases in Finland have increased since 1995 from 2% to more than 5% and from 6% to more than 30%, respectively [11]. Although TB screening including a chest X-ray is recommended for migrants arriving from high-incidence countries, highly infectious pulmonary TB (pTB) cases are emerging among the working age population and exacerbating the risk of TB transmission [11,15].

TB surveillance in Finland relies on the National Infectious Diseases Register (NIDR) [11]. Until 2007, however, only bacteriologically or histologically confirmed cases were registered in the NIDR, while clinically diagnosed cases were not [11]. Achieving bacteriological confirmation from children is challenging and paediatric TB is often a clinical diagnosis [2]. Therefore, the incidence of paediatric TB in Finland has probably been under-reported.

We aimed to compare TB incidence rates in native children under the age of 5 years born during the period with universal vs selective BCG vaccination policy and to assess the epidemiological trends of TB among native and foreign-born children under the age of 15 years in Finland.

## Methods

We conducted a nationwide, population-based, retrospective study of registry data and medical patient records of all active TB cases in children under 15 years of age who were newly diagnosed in Finland between 1995 and 2015.

As a part of national surveillance, it is statutory for clinical microbiology laboratories to notify new *Mycobacterium tuberculosis* isolates and for physicians to notify clinically diagnosed or confirmed TB cases to the NIDR administrated by the National Institute for Health and Welfare (THL) [11]. Until 2006, the NIDR registered only bacteriologically or histologically confirmed cases. After adoption of the standard European Union TB case definition, clinically diagnosed cases have been registered since 2007 [11]. In Finland, children with suspected active TB are admitted for investigations to a public hospital. The Finnish Care Register for Health Care (Hilmo) has registered all public hospitalisations since 1994 with more than 95% coverage [16]. Each visit is classified according to the International Classification of Diseases (ICD)-9 (1987–1995) or ICD-10 (1996–present) [16]. The Finnish Population Information System administrated by the Population Register Centre contains the basic information of residents in Finland. Personal identity codes allow identification of each individual and linkage of data from the NIDR, Hilmo, the Finnish Population Information System and medical patient records.

**Figure 1:**
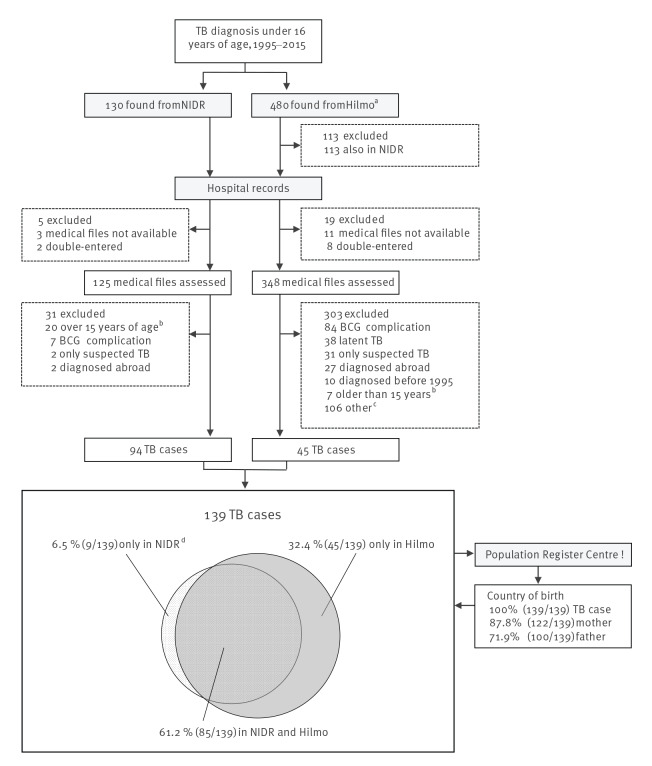
Flowchart of the record linkage from the data sources and exclusion of cases, paediatric tuberculosis, Finland, 1995–2015 (n = 139)

BCG: *Bacillus* Calmette–Guérin; Hilmo: the Finnish Care Register for Health Care; NIDR: the National Infectious Diseases Register; TB: tuberculosis.

^a^ Registered in the Hilmo with any diagnostic code for TB (ICD-9 010–018 or ICD-10 A15–19).

^b^ Age at the time of first consultation for symptoms or of referral that led to the diagnosis.

^c^ Patient without any TB suspicion in medical records (i.e. diagnostic code for TB used by mistake).

^d^ Registered to the Hilmo with other than diagnostic code for TB (ICD-9 010–018 or ICD-10 A15–19).

The data collection flowchart is presented in Figure 1. TB patients under the age of 16 years were identified from the NIDR (accessed 30 June 2016), and patients younger than 16 years with an ICD-9 (010–018) or ICD-10 (A15–19) diagnostic code for TB were identified from the Hilmo (accessed 17 November 2016). We requested the medical patient records of all identified TB patients from the relevant hospitals, verified the TB diagnosis and collected clinical data. We considered cases to be incident at the time of their first consultation for symptoms or of referral that led to the diagnosis, and patients under 15 years of age were classified as paediatric TB cases and included in the data analysis. The patients or their families were not contacted. A case of active TB was defined as a person diagnosed and started on a full course of TB treatment with or without bacteriological or histological confirmation. We excluded patients whose treatment was discontinued because the diagnosis changed to other than active TB, who were initially diagnosed abroad or whose medical records were not available. The National Vaccination Register (NVR) does not include BCG data from the study period. The BCG status was classified from the medical records as vaccinated, non-vaccinated or unknown. The patients’ and parents’ country of birth were obtained from the Population Register Centre. The annual number of TB cases older than 15 years for the overall TB incidence trend was obtained from the open access NIDR database (accessed 18 Oct 2018) [17]. Demographic data of the birth cohorts and population was obtained from the Statistics Finland population database (accessed 18 Oct 2018) [18].

Cases were defined as bacteriologically confirmed or clinically diagnosed, pulmonary or extrapulmonary, and multidrug-resistant according to the WHO guidelines [1]. An index case was defined from the medical records and classified as a household contact if the child had lived in the same household at any time during transmission. Patients were classified as native (born in Finland) or foreign-born. Native cases were further classified as second-generation migrants if at least one parent had been born abroad. According to the national selective BCG guideline published by the THL, the children were classified to be eligible for BCG if the child or at least one parent had been born in a country with high TB incidence, classified as incidence of at least 50 per 100,000 population [19].

### Statistics

For comparison of descriptive data between the native and foreign-born TB cases, we used a Mann–Whitney U and two-tailed Fisher’s exact or chi-squared test for the analysis of continuous and categorical data, respectively. We used a logistic regression model and its predictive margins to calculate proportions of pTB and bacteriologically confirmed TB adjusted for age and/or for site of infection. We calculated the incidence rate of TB in children under 5 years of age per 100,000 person-years in the birth cohorts with universal (1995 to August 2006) and selective vaccination (September 2006 to 2015) and used a Poisson log-linear model to estimate the incidence rate ratio (IRR) between the birth cohorts. We assessed if there was overdispersion with respect to the Poisson model from residual deviance divided by degrees of freedom or using the likelihood ratio test, and assessed if negative binomial regression was needed. We calculated the annual TB incidence rate for all ages, all children under 15-years, native children under 15-years, foreign-born children under 15-years and foreign-born children under 15-years from high-TB incidence countries, and analysed incidence trends in these populations with a negative binomial model with year as the sole explanatory variable. The specific codes for the models are available from the authors. A p value < 0.05 was considered statistically significant. Data were analysed with SPSS Statistics (Version 24, IBM Corporation, Armonk, United States (US)) and Stata15.1 (StataCorp LLC, TX, US). 

### Ethical statement

The procedures described here were carried out in accordance with the ethical standards described in the Helsinki Declaration revised in 2013. The ethics approval for the study was given by the Research Ethical Committee of the THL (THL/1306/5.05.00/2016).

## Results

We identified a total of 139 paediatric TB cases (Figure 1). A total of 67 cases were diagnosed in 2007 to 2015 of whom 11 (16.4%) were only registered in the Hilmo.

The detailed demographics and characteristics of the patients are presented in Table 1. Eligibility for BCG vaccination could be determined conclusively for 138 (99.3%) of the 139 cases; one case born in a low-TB incidence country to a mother from a low-TB incidence country without paternal data was classified as ineligible for BCG. Among the 18 native TB cases born during selective BCG policy, seven were eligible for BCG of whom five were BCG-vaccinated and two had an unknown BCG status. The index case was identified in 64 (46.0%) of the 139 cases. Among the 27 native cases with parents born in a low-TB incidence country, the index case was identified in 21 and a household contact in 11. There was one death due to TB (1/139; 0.7%): an infant born in Finland during the selective BCG policy with native-born parents and not BCG-vaccinated.

**Table 1:** Demographic and clinical characteristics of children under 15 years of age with newly diagnosed active tuberculosis, Finland, 1995–2015 (n = 139)

	All		Native		Foreign-born		p value
	n	%	n	%	n	%	
Tuberculosis cases	139	100	50	36.0	89	64.0	NA
Second-generation migrant	24	17.3	24	48.0	NA	NA	NA
BCG-eligible^a^	109	78.4	23	46.0	86	96.6	**< 0.0001**
Sex, male	72	51.8	25	50.0	47	52.8	0.75
Country of birth^b^							
Somalia	59	42.4	7	14.0	52	58.4	NA
Ethiopia	10	7.2	4	8.0	6	6.7	
Thailand	5	3.6	3	6.0	2	2.2	
Afghanistan	6	4.3	1	2.0	5	5.6	
Other	33	23.4	9	18.0	24	27.0	
Age in years							
Median	9.3		3.7		11.4		**< 0.0001**
IQR	4.1–12.6		1.7–8.4		7.1–13.5		
< 5	40	28.8	28	56.0	12	13.5	**< 0.0001**
5–14	99	71.2	22	44.0	77	86.5	
BCG							
Yes	49	35.3	19	38.0	30	33.7	0.08^c^
No	27	19.4	5	10.0	22	24.7	
Unknown	63	45.3	26	52.0	37	41.6	0.24
Case finding							
Symptoms	67	48.2	21	42.0	46	51.7	**0.002** ^d^
Contact investigation	48	34.5	29	58.0	19	21.3	
TB screening	24	17.3	NA	NA	24	27.0	NA
Index case							
Household contact	45	32.4	19	38.0	26	29.2	**0.0005** ^c^
Other	19	13.7	17	34.0	2	2.2	
Unknown	75	54.0	14	28.0	61	**68.5**	**< 0.0001**
Clinical characteristics							
pTB	75	54.0	31	62.0	44	49.4	0.42^e^
Smear-positive pTB^g^	8	10.7	3	9.7	5	11.4	0.99
Bacteriologically confirmed	63	45.3	16	32.0	47	52.8	0.15^f^
MDR-TB	1	0.7	0	0	1	1.1	0.99

BCG: *Bacillus* Calmette–Guérin; IQR: interquartile range; MDR: multidrug-resistant; NA, not applicable; pTB: pulmonary tuberculosis; TB: tuberculosis.

^a^ Patient or parent(s) born in a high-TB incidence country (≥ 50/100,000 population).

^b^ Birth country of the child if foreign-born (n = 89) or of the parent(s) if second-generation migrant (n = 24). Native children with Finnish born parents (n = 26) not shown.

^c^ Unknown excluded from the analysis.

^d^ TB screening excluded from the analysis.

^e^ Adjusted for age.

^f^ Adjusted for age and infection focus (pulmonary or non-pulmonary).

^g^ Denominator: patients for whom at least one respiratory smear sample obtained.

Bold numbers indicate statistical significance at p < 0.05. Percentages represent row or column percentages.

**Figure 2:**
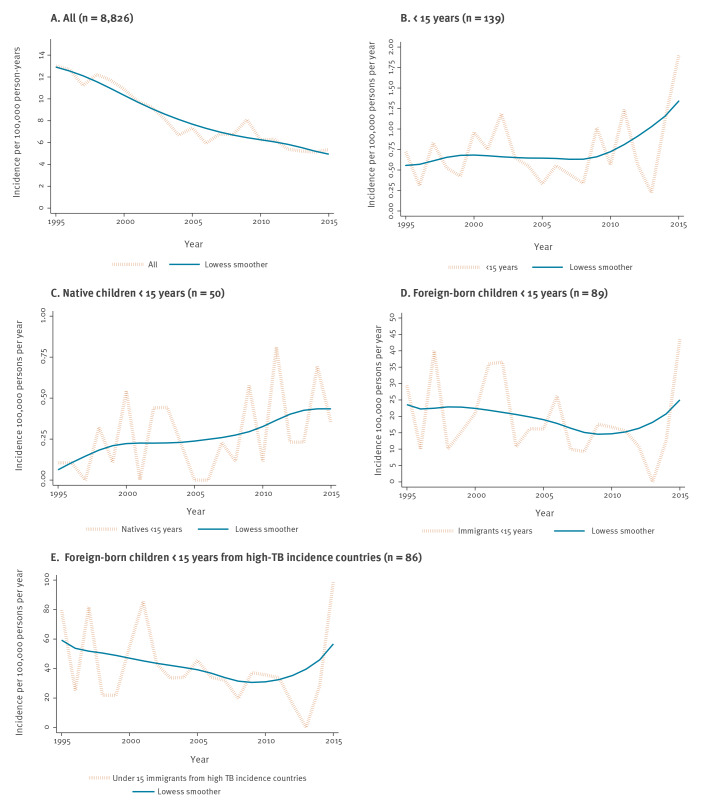
Annual incidence of tuberculosis in Finland, 1995–2015

The demographics of the population in Finland and annual incidence of TB in the period 1995 to 2015 are listed in the Supplementary Table S1. The TB incidence rate trends from 1995 to 2015 are presented in Figure 2. During this period, the overall TB rate in Finland showed a decreasing trend (IRR = 0.95; 95% CI: 0.95–0.96). The TB rate among children under the age of 15 years – all vs foreign-born vs foreign-born from high-TB incidence countries – did not change significantly (IRR = 1.02; 95% CI: 0.99–1.06 vs IRR = 0.98; 95% CI: 0.94–1.02 vs IRR = 0.98; 95% CI: 0.94–1.02). The TB rate among native children younger than 15 years showed a slightly increasing trend with a relative annual increase of 6% (IRR = 1.06; 95% CI: 1.01–1.11).

TB: tuberculosis.

Lowess, locally weighted scatterplot smoothing. The scale along the y-axis for the associated incidence value varies by plot.

In the period 1995 to 2015, a total of 1,227,221 children were born in Finland. The country of birth of the mother was available for 99.8%. The 1995 to 2015 birth cohorts and the number of TB cases in each birth cohort are detailed in Table 2. The cohorts born during the universal and the selective BCG period comprised, respectively, 678,221 children observed for 3,391,105 person-years and 549,000 children observed for 2,030,509 person-years. The IRR of TB in children younger than 5 years in the selective-BCG cohorts compared with the universal-BCG cohorts was 1.3 (95% CI: 0.7–2.3; p = 0.47). There were two cases of severe TB: one born in 2004 during universal BCG policy and one born in 2009 during the selective BCG policy.

**Table 2:** Birth cohorts born during universal or selective BCG vaccination policy and incidence of active tuberculosis in those younger than 5 years, per 100,000 person-years in each cohort, Finland, 1995–2015 (n = 42)

Birth cohort	Cohort population	Foreign-born mother		High-risk mother^a^		Total person-years^b^	TB cases (severe^c^)		Total incidence rate^e^	
		n	%	n	%		Total	With a high-risk parent^d^	Incidence	95% CI
Universal BCG policy										
1995	63,067	1,881	3.0	1,179	1.9	315,335	4	2	1.3	0.3–3.2
1996	60,723	1,989	3.3	1,248	2.1	303,615	4	4	1.3	0.4–3.4
1997	59,329	2,133	3.6	1,371	2.3	296,645	1	1	0.3	0.01–1.9
1998	57,108	2,267	4.0	1,439	2.5	285,540	0	0	0.0	0.0–1.3
1999	57,574	2,382	4.1	1,536	2.7	287,870	2	2	0.7	0.1–2.5
2000	56,742	2,381	4.2	1,560	2.7	283,710	3	1	1.1	0.2–3.1
2001	56,189	2,633	4.7	1,700	3.0	280,945	2	0	0.7	0.1–2.6
2002	55,555	2,696	4.9	1,777	3.2	277,775	0	0	0.0	0.0–1.3
2003	56,630	2,825	5.0	1,883	3.3	283,150	3	1	1.1	0.2–3.1
2004	57,758	2,959	5.1	1,942	3.4	288,790	2 (1)	2 (1)	0.7	0.1–2.5
2005	57,745	3,220	5.6	2,104	3.6	288,725	1	0	0.3	0.01–1.9
I/2006^f^	39,801	2,378	6.0	NA	NA	199,005	2	1	1.0	0.1–3.6
Selective BCG policy										
II/2006^g^	19,039	1,138	6.0	NA	NA	95,195	2	1	2.1	0.3–7.6
2007	58,729	3,690	6.3	2,417	4.1	293,645	3	0	1.0	0.2–3.0
2008	59,530	3,923	6.6	2,614	4.4	297,650	4	2	1.3	0.4–3.4
2009	60,430	4,290	7.1	2,806	4.6	302,150	2 (1)	1 (0)	0.7	0.1–2.4
2010	60,980	4,760	7.8	3,071	5.0	304,900	1	0	0.3	0.01–1.8
2011	59,961	4,969	8.3	3,300	5.5	269,825	2	0	0.7	0.1–2.7
2012	59,493	5,415	9.1	3,594	6.0	208,226	1	0	0.5	0.01–2.7
2013	58,134	5,625	9.7	3,691	6.3	145,335	1	1	0.7	0.02–3.8
2014	57,232	6,219	10.9	4,069	7.1	85,848	2	2	2.3	0.3–8.4
2015	55,472	6,363	11.5	4,195	7.6	27,736	0	0	0.0	0.0–13.3

BCG: *Bacillus* Calmette–Guérin; CI: confidence interval; NA: not available; TB: tuberculosis.

^a^ Mother born in a high-TB incidence country (≥ 50/100,000 population).

^b^ Total person-years in cohort observed until 31 December 2015 or in those 5 years of age.

^c^ Meningeal or miliary.

^d^ At least one parent born in a high-TB incidence country (≥ 50/100,000 population).

^e^ Number of cases per 100,000 person-years.

^f^ Born from January to August 2006.

^g^ Born from September to December 2006.

## Discussion

Nine years into the selective BCG policy in Finland, the incidence of TB or severe TB in native children under the age of 5 years has not increased. The first years of life are the most critical without the protection of BCG: young children are vulnerable to severe TB and benefit from BCG immunisations the most [2,4]. A transient increase in TB and severe disease in those younger than 5 years has previously been reported from Sweden and the Czech Republic after a shift from universal to selective BCG vaccinations [9,10]. In France, universal BCG vaccinations were discontinued in 2007, with seemingly very little impact on the incidence of paediatric TB or TB meningitis [20,21]. Multiple factors might explain why also in Finland, TB morbidity at age under 5 years did not increase: the TB incidence in the general population at the time of the BCG policy change was lower and the TB burden was concentrated to the indigenous older population [12]. Capturing the target group for selective vaccinations is also decisive yet unpredictable [8,9,22]. In Finland, the implementation of selective vaccinations was planned meticulously and BCG eligibility is determined in advance at public maternity clinics with very high attendance [12]. Thus, the immediate coverage of the target population was probably high. Nevertheless, as adult TB remains uneradicated, TB exposure and infections among young children are inevitable. With the majority of the infant population not BCG-vaccinated, identifying TB-infected young children quickly through other TB control measures becomes increasingly imperative. 

While the overall TB rate in Finland continues to decline, the incidence of TB in the population younger than 15 years is not decreasing. In fact, the incidence of TB in the native population younger than 15 years, including children born during both BCG policy periods, showed a slightly increasing trend. Simultaneously, the demographics in Finland have changed considerably. It is likely that the observed trends reflect an increasing proportion of children under 15 years with second-generation migrant background and a higher TB risk than those with Finnish-born parents; this is further demonstrated by the sudden increase in asylum seekers and subsequent migrant TB cases in 2015 [23]. The TB incidence under 15 years in Finland was expected to increase slightly following the BCG policy revision [13,24]. Evaluation of the impact of the decreased BCG coverage on TB morbidity in that age group is possible when the selective BCG cohorts grow older.

Paediatric TB morbidity in Finland parallels the status in other parts of northwestern Europe: it is concentrated to families with an migrant background from TB-endemic countries [9,25,26]. The index of a non-native case was frequently unknown, suggesting that foreign-born children are either exposed to TB abroad before arriving in Finland or their index cases in Finland remain unidentified. Furthermore, most foreign-born children were diagnosed through symptoms and less than a third through migrant TB screening, suggesting that TB screening and/or contact investigations seem to miss foreign-born children with TB infections. The usual presentation was pTB; extrapulmonary disease was more common among foreign-born children but the difference was insignificant after adjusting for age. Approximately 11% of pTB cases were smear-positive, suggesting a low transmission risk of paediatric TB. Clinical diagnoses were common, highlighting the shortcomings of the current diagnostic techniques for paediatric TB. The proportion confirmed bacteriologically was higher among foreign-born children but the difference was insignificant after adjusting for age and site of infection. Our smear-positive rate compares well with previous studies [27]. The proportions of pTB and bacteriologically confirmed TB show a similar tendency as recent study from the United Kingdom (UK): young children are more likely to have pTB, and bacteriological confirmation is less likely among young, native TB cases [25]. 

Selective BCG vaccinations depend strongly on the identification and capture of children with higher risk of TB exposure. In Finland, the estimated TB reduction with BCG immunisations of risk groups with a TB incidence ≥ 23.7/100,000 is close to that achieved with universal BCG [13]. The TB rate among the migrant population under 15 years from high-TB incidence countries was well above this threshold. Furthermore, 23 of the 50 native-born TB cases had a parent(s) born in a country with high TB incidence, suggesting that a higher TB exposure risk passes to the second generation. Many native cases, however, had no evident parental TB exposure risk and yet had an index case within the household. In Finland, the older indigenous population (e.g. grandparents) remains a major reservoir for TB reactivation; they contracted TB infections when TB was endemic in their youth and still account for the majority of all TB cases [11,15]. This is likely to cause transmission risk directly to the grandchildren or indirectly through the parents. Inevitably, some high-risk children who would have been captured with universal BCG vaccination will remain unvaccinated under the selective policy. BCG status was unknown for most native TB cases, although most had been born during the universal policy and were likely to be vaccinated. We did not identify any BCG-eligible native TB cases born during the selective policy who were unvaccinated, although two had an unknown BCG status. In the future, the NVR can further clarify the capture and weaknesses of our current selective vaccinations.

Accurate paediatric TB surveillance data are essential for evaluating the success of BCG policy. The NIDR is the only source for national TB data reported in Finland and in 2007, the estimated under-reporting was 5% [28]. Overall, the NIDR was missing 32% of paediatric TB cases: most were clinically diagnosed before 2007 and probably missing because of the former strict register criteria. However, the NIDR was also missing 11 of the 67 paediatric TB cases diagnosed between 2007 and 2015. Evidently, relying on notifications from physicians can cause a major gap in case detection resulting in under-reporting of paediatric TB morbidity. Undernotification of paediatric TB from high-burden countries has become increasingly acknowledged [29]. However, statutory notifications are considered a good proxy indication of the true incidence in most of Europe [1]. Our findings compare well with the UK where an estimated 20% of paediatric cases were missing from the national surveillance system [30]. Although published figures on TB under-reporting in Europe generally lack estimates specifically for the paediatric population, data suggest that culture- or smear-negative TB cases are more likely to remain unnotified [31,32]. Since achieving bacteriological confirmation in children is challenging, it is likely that compared with adults, paediatric TB cases are more often unnotified across Europe [2,31,32]. The World Health Organization adjustment factor accounting for under-reporting is standard for most European TB surveillance data [1]. Separate adjustment factors would probably increase the reliability of the estimates and, therefore, further inventory studies focusing on paediatric TB in Europe should be encouraged.

Our study had some limitations and the results should be interpreted with caution. All TB cases might not be captured, and the number of cases was small. Comprehensive BCG coverage of the patients or birth cohorts was not available. Index cases were based on the epidemiological link rather than molecular epidemiology. Although we did not observe an increasing trend for the TB rate in native children younger than 5 years, minor changes might be concealed under the overall decreasing TB trend in Finland or not achieve statistical significance.

## Conclusion

We portray a rare view into paediatric TB epidemiology: a nationwide study looking at a 21-year period including a transition into a low-incidence country along with a major demographic shift and fundamental revision of the BCG policy. A major strength of our study is the capture of paediatric TB cases from separate nationwide registers, with all cases confirmed from the medical patient records. The paediatric TB morbidity in Finland is concentrated in families with an migrant background from TB endemic countries. The TB morbidity in native children younger than 5 years in Finland did not increase after the BCG policy revision, suggesting that well implemented selective vaccinations can prevent TB in the most vulnerable age group nearly as effectively as universal vaccinations in low-incidence settings. Even in a high-income country with statutory notifications, the national TB surveillance registry can miss a substantial number of paediatric TB cases. Implementing separate adjustment factors for paediatric TB notifications could increase the reliability of TB surveillance data.

## Supplementary Data

173000 Supplement
