# Correction for Euro Surveill. 2020;26(11)

**DOI:** 10.2807/1560-7917.ES.2021.26.15.210415c

**Published:** 2021-04-15

**Authors:** Eurosurveillance editorial team

**Affiliations:** 1 European Centre for Disease Prevention and Control (ECDC), Stockholm, Sweden

In the article ‘Paediatric tuberculosis during universal and selective Bacillus Calmette–Guérin vaccination policy: a nationwide population-based retrospective study, Finland, 1995–2015’ by Kontturi et al., published on 18 March 2021, a statement regarding funding was added to the acknowledgements’ section on 12 April 2021, at the request of the authors.

