# Supplementary material for: Paediatric tuberculosis during universal and selective Bacillus Calmette–Guérin vaccination policy: a nationwide population-based retrospective study, Finland, 1995–2015
Source: Euro Surveill. 2021 Mar 18;26(11):1900711. doi: 10.2807/1560-7917.ES.2021.26.11.1900711 (PMC7976386; doi:10.2807/1560-7917.ES.2021.26.11.1900711)
Supplement: Supplement [file 19-00711_KONTTURI_Supplementary-Table.docx]

**This supplementary material is hosted by Eurosurveillance as supporting information alongside the article *Paediatric tuberculosis during universal and selective Bacille Calmette-Guérin vaccination policy: a nationwide population based retrospective study, Finland, 1995–2015*, on behalf of the authors, who remain responsible for the accuracy and appropriateness of the content. The same standards for ethics, copyright, attributions and permissions as for the article apply. Supplements are not edited by Eurosurveillance and the journal is not responsible for the maintenance of any links or email addresses provided therein.**

**Supplementary table 1.**

| **Year** | **All** | | | | **Under 15** | | | **Under 15 natives** | | | **Under 15 immigrants** | | | **Under 15 immigrants from high TB incidence country**^f^ | | | |
| --- | --- | --- | --- | --- | --- | --- | --- | --- | --- | --- | --- | --- | --- | --- | --- | --- | --- |
|  | **Populatio**^a^ | **% immigrants**^a^ | **TB**  **cases**^b^ | **Incidence**  **(95% CI)**^c^ | **Population**^a^ | **TB cases** | **Incidence**  **(95% CI)**^c^ | **Population**^a^ | **TB cases** | **Incidence**  **(95% CI)**^c^ | **Population**^a,d^ | **TB cases** | **Incidence**  **(95% CI)**^c^ | **Population^a,e^** | **TB cases** | **Incidence**  **(95% CI)**^c^ | |
| 1995 | 5010523 | 2.1 | 651 | 13.0 (12.0–14.0) | 971770 | 7 | 0.7 (0.3–1.5) | 951329 | 1 | 0.1 (0.003–0.6) | 20441 | 6 | 29.4 (10.8–63.9) | 7553 | 6 | 79.4 (29.2–172.9) | |
| 1996 | 5021189 | 2.2 | 637 | 12.7 (11.7–13.7) | 968567 | 3 | 0.3 (0.1–0.9) | 948549 | 1 | 0.1 (0.003–0.6) | 20018 | 2 | 10.0 (1.2–36.1) | 7946 | 2 | 25.2 (3.0–90.9) | |
| 1997 | 5029279 | 2.3 | 564 | 11.2 (10.3–12.2) | 961350 | 8 | 0.8 (0.4–1.6) | 941353 | 0 | 0.0 (0.0–0.4) | 19997 | 8 | 40.0 (17.3–78.8) | 8556 | 7 | 81.8 (32.9–168.6) | |
| 1998 | 5034596 | 2.5 | 616 | 12.2 (11.3–13.2) | 951145 | 5 | 0.5 (0.2–1.2) | 931238 | 3 | 0.3 (0.1–0.9) | 19907 | 2 | 10.0 (1.2–36.3) | 9107 | 2 | 22.0 (2.7–79.3) | |
| 1999 | 5040182 | 2.6 | 591 | 11.7 (10.8–12.7) | 943001 | 4 | 0.4 (0.1–1.1) | 923477 | 1 | 0.1 (0.003–0.6) | 19524 | 3 | 15.4 (3.2–44.9) | 9136 | 2 | 21.9 (2.7–79.1) | |
| 2000 | 5044912 | 2.7 | 549 | 10.9 (10.0–11.8) | 936333 | 9 | 1.0 (0.4–1.8) | 917219 | 5 | 0.5 (0.2–1.3) | 19114 | 4 | 20.9 (5.7–53.6) | 9088 | 4 | 55.0 (17.9–128.4) | |
| 2001 | 5049766 | 2.9 | 493 | 9.8 (8.9–10.7) | 931587 | 7 | 0.8 (0.3–1.5) | 912151 | 0 | 0.0 (0.0–0.4) | 19436 | 7 | 36.0 (14.5–74.2) | 9352 | 7 | 85.5 (36.9–168.6) | |
| 2002 | 5054238 | 3.0 | 468 | 9.3 (8.4–10.1) | 927009 | 11 | 1.2 (0.6–2.1) | 907850 | 4 | 0.4 (0.1–1.1) | 19159 | 7 | 36.5 (14.7–75.3) | 9284 | 7 | 43.1 (11.7–110.3) | |
| 2003 | 5060865 | 3.1 | 411 | 8.1 (7.4–8.9) | 920097 | 6 | 0.7 (0.2–1.4) | 901492 | 4 | 0.4 (0.1–1.1) | 18605 | 2 | 10.7 (1.3–38.8) | 8939 | 2 | 33.6 (6.9–98.1) | |
| 2004 | 5070250 | 3.3 | 338 | 6.7 (6.0–7.4) | 914560 | 5 | 0.5 (0.2–1.3) | 896075 | 2 | 0.2 (0.03–0.8) | 18485 | 3 | 16.2 (3.3–47.4) | 8805 | 3 | 34.1 (7.0–99.6) | |
| 2005 | 5078968 | 3.5 | 373 | 7.3 (6.6–8.1) | 906904 | 3 | 0.3 (0.1–1.0) | 888302 | 0 | 0.0 (0.0–0.4) | 18602 | 3 | 16.1 (3.3–47.1) | 8777 | 3 | 45.6 (12.4–116.7) | |
| 2006 | 5089045 | 3.7 | 301 | 5.9 (5.3–6.6) | 901181 | 5 | 0.6 (0.2–1.3) | 882116 | 0 | 0.0 (0.0–0.4) | 19065 | 5 | 26.2 (8.5–61.2) | 8805 | 5 | 34.1 (7.0–99.6) | |
| 2007 | 5097956 | 4.0 | 346 | 6.8 (6.1–7.5) | 894590 | 4 | 0.4 (0.1–1.1) | 874507 | 2 | 0.2 (0.03–0.8) | 20083 | 2 | 10.0 (1.2–36.0) | 9332 | 2 | 32.2 (6.6–93.9) | |
| 2008 | 5107688 | 4.3 | 343 | 6.7 (6.0–7.5) | 891162 | 3 | 0.3 (0.1–1.0) | 869589 | 1 | 0.1 (0.003–0.6) | 21573 | 2 | 9.3 (1.1–33.5) | 10104 | 2 | 19.8 (2.4–71.5) | |
| 2009 | 5118244 | 4.6 | 415 | 8.1 (7.3–8.9) | 888323 | 9 | 1.0 (0.5–1.9) | 865555 | 5 | 0.6 (0.2–1.3) | 22768 | 4 | 17.6 (4.8–45.0) | 10755 | 4 | 37.2 (10.1–95.2) | |
| 2010 | 5127141 | 4.8 | 320 | 6.2 (5.6–7.0) | 887677 | 5 | 0.6 (0.2–1.3) | 863798 | 1 | 0.1 (0.003–0.6) | 23879 | 4 | 16.8 (4.6–42.9) | 11182 | 4 | 35.8 (9.7–91.6) | |
| 2011 | 5135119 | 5.2 | 324 | 6.3 (5.6–7.0) | 888982 | 11 | 1.2 (0.6–2.2) | 863308 | 7 | 0.8 (0.3–1.7) | 25674 | 4 | 15.6 (4.2–39.9) | 11851 | 4 | 33.8 (9.2–86.4) | |
| 2012 | 5141203 | 5.6 | 277 | 5.4 (4.8–6.1) | 891392 | 5 | 0.6 (0.2–1.3) | 863544 | 2 | 0.2 (0.03–0.8) | 27848 | 3 | 10.8 (2.2–31.5) | 12540 | 2 | 16.0 (1.9–57.6) | |
| 2013 | 5146991 | 5.9 | 269 | 5.2 (4.6–5.9) | 895021 | 2 | 0.2 (0.03–0.8) | 865126 | 2 | 0.2 (0.03–0.8) | 29895 | 0 | 0.0 (0.0–12.3) | 13244 | 0 | 0.0 (0.0–27.9) | |
| 2014 | 5149776 | 6.3 | 264 | 5.1 (4.5–5.8) | 896608 | 10 | 1.1 (0.5–2.1) | 864993 | 6 | 0.7 (0.3–1.5) | 31615 | 4 | 12.7 (3.4–32.4) | 13944 | 4 | 28.7 (7.8–73.4) | |
| 2015 | 5150146 | 6.5 | 276 | 5.4 (4.7–6.0) | 896023 | 17 | 1.9 (1.1–3.0) | 863838 | 3 | 0.3 (0.1–1.0) | 32185 | 14 | 43.5 (23.8–73.0) | 14132 | 14 | 99.1 (54.2–166.2) | |
| Total | – | – | 8826 | – | – | 139 | – | – | 50 | – | – | 89 | – | – | 86 | – | |
| **Note.**  CI, Confidence interval.  ^a^ Statistics Finland (www.stat.fi/, accessed Oct 18, 2018).  ^b^ The National Infectious Diseases Register (www.thl.fi/ttr/gen/rpt/tilastot.html, accessed Oct 18, 2018) with missing TB cases of children under 15 years of age added.  ^c^ Cases per 100,000 person years.  ^d^ Those with an unknown birth country included.  ^e^ Those with an unknown birth country excluded.  ^f^ TB incidence ≥50/100,000 population | | | | | | | | | | | | | | | | |  |

Annual active TB incidence per 100,000 in different populations in Finland, 1995–2015.
